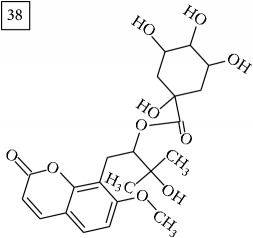# Changes of Metabolomic Profile in* Helianthus annuus* under Exposure to Chromium(VI) Studied by capHPLC-ESI-QTOF-MS and MS/MS

**DOI:** 10.1155/2017/3568621

**Published:** 2017-11-22

**Authors:** Alan Alexander Gonzalez Ibarra, Kazimierz Wrobel, Eunice Yanez Barrientos, Alma Rosa Corrales Escobosa, J. Felix Gutierrez Corona, Israel Enciso Donis, Katarzyna Wrobel

**Affiliations:** ^1^Chemistry Department, University of Guanajuato, L. de Retana 5, 36000 Guanajuato, GTO, Mexico; ^2^Biology Department, University of Guanajuato, L. de Retana 5, 36000 Guanajuato, GTO, Mexico

## Abstract

The application of capHPLC-ESI-QTOF-MS and MS/MS to study the impact of Cr(VI) on metabolites profile in* Helianthus annuus *is reported. Germinated seeds were grown hydroponically in the presence of Cr(VI) (25 mgCr/L) and root extracts of the exposed and control plants were analyzed by untargeted metabolomic approach. The main goal was to detect which metabolite groups were mostly affected by Cr(VI) stress; two data analysis tools (ProfileAnalysis, Bruker, and online XCMS) were used under criteria of intensity threshold 5 · 10^4^ cps, fold change ≥ 5, and *p* ≤ 0.01, yielding precursor ions. Molecular formulas were assigned based on data processing with two computational tools (SIRIUS and MS-Finder); annotation of candidate structures was performed by database search using CSI:FingerID and MS-Finder. Even though ultimate identification has not been achieved, it was demonstrated that secondary metabolism became activated under Cr(VI) stress. Among 42 candidate compounds returned from database search for seven molecular formulas, ten structures corresponded to isocoumarin derivatives and eleven were sesquiterpenes or sesquiterpene lactones; three benzofurans and four glycoside or pyrane derivatives of phenolic compounds were also suggested. To gain further insight on the effect of Cr(VI) in sunflower, isocoumarins and sesquiterpenes were selected as the target compounds for future study.

## 1. Introduction

There are several types of abiotic stress affecting plants and metabolomic tools have often been used to investigate plant response or tolerance [1–3]. Some metal/metalloid stressors, such as cadmium, copper or arsenic, received considerable attention [2, 4–6] whereas metabolomic approach in studies of hexavalent chromium (Cr(VI)) has been scarce [7]. Specifically, metabolic response in rice roots was evaluated using nuclear magnetic resonance (NMR) and gas chromatography-mass spectrometry (GC-MS) [7]. In another work, liquid chromatography-high resolution mass spectrometry (HPLC-HRMS) was utilized for untargeted metabolomics in wild and transgenic* Nicotiana langsdorffii* under exposure to Cr(VI) [8].

The uptake, distribution, speciation, and toxic effects of Cr(VI) in different plant species are well documented [9, 10] and, for* Helianthus annuus,* its feasibility for phytoremediation purposes has been demonstrated [11, 12]. Therefore, it seemed relevant to evaluate the impact of Cr(VI) on metabolite profile in this specific plant.

Between two main analytical platforms in use, mass spectrometry based procedures present higher sensitivity and higher throughput in the identification of multiple metabolites in biological matrices with respect to NMR [13]. When liquid chromatography is coupled to HRMS, information on ionic or thermally unstable compounds can be obtained upon simple sample pretreatment and without precolumn derivatization; however, raw data are highly complex and, due to the variety of chromatographic/ionization conditions available, metabolites identification by means of database search is not so straightforward as for GC-MS [14].

As to the instrumental setup of LC-MS, column effluent is usually introduced via electrospray ionization (ESI) to a time of flight (TOF), quadrupole-time of flight (QTOF), linear trap quadrupole-Orbitrap (LTQ-Orbitrap), or Fourier transform ion cyclotron resonance (FT-ICR) high resolution mass spectrometer. For the determination of specific metabolites (targeted metabolomics), triple quadrupole (QqQ) mass filter is highly indicated [15]. Multidimensional LC-MS raw data require extensive processing and both identification of compounds and extraction of biologically relevant information rely on chemometric tools. In a typical workflow of data preprocessing, noise is filtered and background corrected; then peaks are detected, deconvoluted, aligned, and normalized yielding a list of molecular features [16]. In untargeted metabolomics, this is a starting point for metabolite identification. If the purpose is to compare two biological conditions (exposed to Cr(VI) versus nonexposed plants), statistical tools can be used for detecting fold changes of individual signals under established statistical significance criterion. This latter procedure substantially decreases the number of compounds to be identified; the list of precursor ions is defined and, in the additional chromatographic run, MS/MS spectra are acquired [3]. Data preprocessing and statistical analysis can be carried out using software packages provided by instrument manufacturer; such is the case of DataAnalysis and ProfileAnalysis, respectively, for Bruker Daltonics spectrometers. On the other hand, several computational platforms, ready to use for raw data from different instruments, are available; in this work XCMS (https://xcmsonline.scripps.edu) was applied [17, 18].

Analysis of crude extracts containing different metabolite types is of interest while evaluating Cr(VI) impact on plant metabolome although, in such untargeted approach, annotation and identification of individual compounds are extremely difficult. The main reason for this situation is a large variety of metabolites with similar molecular masses, structures, and functionalities so even the mass accuracy error < 1 ppm is far from guaranteeing definitive compound identification [19], the limitation especially important while characterizing secondary metabolites involved in plant response to stress. Another difficulty is related to the low metabolite coverage in the existing databases; according to the recent estimation, 50899 structures included in KNApSAcK database of plant metabolites (http://kanaya.naist.jp/KNApSAcK/) correspond only to about 5% of all known metabolites [20]. Finally, metabolites are usually present in a wide concentration range and detection of low abundance signals is problematic. Therefore, annotation has often been limited to certain classes of compounds and differentiation among isomers could hardly be achieved.

Improving the identification power in untargeted metabolomics still is one of the most challenging research areas. The following data are needed for identification based on LC-MS analysis: retention time; formation of adducts; exact mass of the precursor ion; isotopic pattern derived from relative isotopic abundance of individual elements composing the molecule and fragmentation spectra. Several computational methods have been developed, based on one or more of the above factors. Unrevealing the exact mass is always the first step, which is followed by generation of molecular formula, database search of candidate compounds, and elucidation of molecular structure from MS/MS spectra. To mention some tools used in the present work, SIRIUS (https://bio.informatik.uni-jena.de/sirius2/) utilizes isotopic patterns acquired by HRMS and generates the fragmentation trees; structural elucidation of metabolite is based on the statistical comparison of experimental spectra with those obtained in silico [21]. Another option is MS-Finder which predicts metabolite formula from experimental MS and MS/MS spectra while applying a series of heuristic rules and a database for neutral losses; the obtained candidates are ranked based on the statistical criteria of matching (http://prime.psc.riken.jp/) [13, 20].

The goal of this work was to find differences in the metabolomic profile of sunflower under Cr(VI) exposure with respect to the nonexposed plants and to apply some of the available chemometric tools for characterization of metabolites involved in plants response. Based on data preprocessing and statistical tests carried out by XCMS and ProfileAnalysis, the precursors list was generated. Application of SIRIUS and MS-Finder tools enabled enhanced reliability during annotation of molecular formulas and were helpful in assignation of candidate compounds to specific groups of secondary metabolites with only few molecular structures of high score found per each molecular formula. The results obtained indicate increased synthesis of biologically active isocoumarins and sesquiterpene lactones in response to Cr(VI) stress in* Helianthus annuus*.

## 2. Materials and Methods

### 2.1. Reagents

All chemicals were of analytical reagent grade. Deionized water (18.2 M*Ω* cm, Labconco, USA), LC-MS-grade methanol, and acetonitrile (MeCN) from Sigma (Milwaukee, USA) were used throughout.

The following Sigma-Aldrich reagents were used: potassium dichromate (Cr(VI)), formic acid, nitric acid, hydrogen peroxide, and sodium hypochlorite. Stock standard solution of chromium (1000 mg/L) was from Sigma and inductively coupled plasma mass spectrometry (ICP-MS) internal standard mix was from Agilent Technologies.

Hoagland's nutrient solution containing calcium nitrate 0.35 mM, calcium chloride 2.1 mM, magnesium sulfate 0.91 mM, monobasic potassium phosphate 0.97 mM, potassium nitrate 1.22 mM, boric acid 23 *μ*M, manganese chloride 3.9 *μ*M, molybdenum trioxide 23 *μ*M, ferric nitrate 10 *μ*M, zinc nitrate 0.6 *μ*M, and copper sulfate 0.44 *μ*M, pH 5.8, was prepared from Sigma reagents [22].

Sunflower seeds (*Helianthus annuus* L.) were purchased at a local garden market as a product of Vita company, distributed in Mexico by Rancho de Molinos, S.A. de C.V.

### 2.2. Plant Growth

Sunflower seeds were surface-sterilized with 3% m/v sodium hypochlorite for 20 min, washed with deionized water, and then germinated in Petri plates using Whatman filters soaked with Hoagland solution. After five days, seedlings were removed carefully and divided into four groups: two of them were hydroponically grown in Hoagland solution amended with Cr(VI), 25 mgCr/L, and the other two were grown as the controls, without Cr(VI) addition. Plants were harvested after ten days, and roots were separated from aerial parts and pooled separately in eight groups (two biological replicates for each of the following: exposed roots, nonexposed roots, exposed aerial parts, and nonexposed aerial parts). Each biomass was homogenized immediately by grinding in liquid nitrogen and was freeze-dried.

### 2.3. Chromium Determination by ICP-MS

All freeze-dried samples were analyzed. Microwave-assisted acid digestion was performed using 50 mg aliquot of the sample to which 800 *µ*L of deionized water, 200 *µ*L of internal standard solution (2 mg/L each of In, Y, Bi, and Rh; 5 mg/L of Sc; and 10 mg/L of Li), and 1 mL of concentrated nitric acid were added. The samples were heated using the following program: temperature: 180°C, ramp time: 3 min, hold time: 3 min, pressure: 300 psi, power: 300, and stirring: medium (microwave digestion system Discover SP-D; CEM). The samples were centrifuged (13,000*g*, 10 min), and 200 *µ*L portions were 20-fold diluted with deionized water and introduced to the ICP-MS system. An inductively coupled plasma mass spectrometer (Model 7500ce; Agilent Technologies) with a Meinhard nebulizer and Peltier-cooled spray chamber (2°C) was used with the previously reported instrumental operating conditions [23]. The isotopes ^52^Cr and ^53^Cr were monitored and standardized to ^89^Y signals. Calibration was performed with Agilent commercial standard at chromium concentrations of 0, 0.4, 1.0, 5.0, 10, 50, and 100 *µ*g/L and with the internal standards, Y 10 *µ*g/L. The chromium instrumental detection limit was 23 ng/L; method detection limit 19 ng/g was evaluated using 20 times diluted digest of control root biomass [24]. For accuracy checking, NIST 1572 Citrus Leaves certified reference material was analyzed. Chromium concentration found in triplicate analysis of this reference materials was 0.77 ± 0.4 *µ*g/g, in agreement with the certified value of 0.8 ± 0.2 *µ*g/g.

### 2.4. capHPLC-ESI-QTOF-MS and MS/MS Analysis of Root Extracts

The samples analyzed were two biological replicates of the exposed and nonexposed roots (four samples). For metabolites extraction, 1 mL of 80% v/v methanol was added to 25 mg of roots biomass and the mixture was ultrasonicated for 15 min and diluted with deionized water to reach 12% v/v methanol. The samples were centrifuged (13,000*g*, 10 min) prior to their on-column injection.

A mass spectrometer maXis impact ESI-QTOF-MS equipped with DataAnalysis 4.1 (Bruker Daltonics) was coupled to Ultimate 3000 RLSCnano system operated by Hystar 3.2 software (Thermo Scientific Dionex). An Agilent capillary trap (5 × 0.3 mm, C18, 5 *μ*m), a reversed phase capillary column Halo C18 (150 × 0.3 mm, 2.7 *μ*m), and connection capillaries nanoViper (i.d. 50 *µ*m) were used. Two mobile phases were (A) 0.1% v/v aqueous formic acid and (B) 0.1% v/v formic acid in acetonitrile. Keeping the sampler temperature at 4°C, 5 *μ*L of plant extract was loaded on the capillary trap at a flow rate 15 *μ*L/min, using 10% B. After 2 min, the flow was switched to the capillary column maintained at 40°C and the separation was carried out at a flow rate 3 *μ*L/min using the following elution program: 0–54 min linear gradient from 10% to 95% B; 54–56 min, 95% B; 56-57 min, 10% B; finally, 11 min washing with 10% B was applied for column reequilibration which resulted in total chromatographic run of 68 min. The column exit was connected to ESI source using the lock-mass standard* m/z* 299.2945 (methyl stearate) in the ion source. ESI was operated in positive mode with ion spray voltage 4500 V, end plate offset 500 V, dry gas 4 L/min, drying temperature 180°C, and nebulizing gas pressure 0.4 bar. The chromatograms were obtained with acquisition rate 4 Hz for MS within the* m/z* range 50–1250. For the selected precursor list, chromatographic run was repeated using injection volume of 10 *μ*L and MS/MS mode (collision energy 20 eV).

### 2.5. Data Analysis

**Figure 1:**
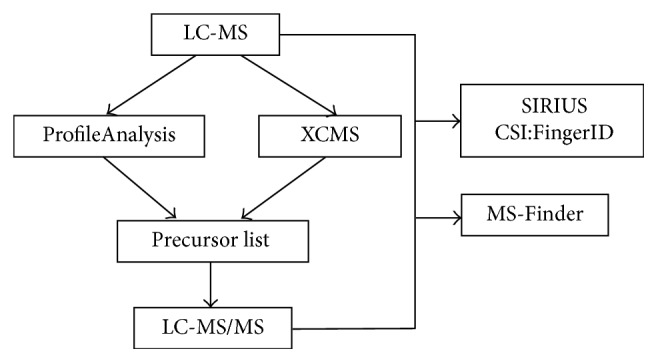
General scheme showing the workflow of data analysis.

A general scheme of data analysis is presented in Figure 1. In the first place, raw capHPLC-ESI-QTOF-MS data acquired for each sample were preprocessed using Bruker DataAnalysis 4.1 which included recalibration of mass accuracy, background subtraction, and finding molecular features (FMF). For FMF, S/N threshold of 3, minimum compound length of 20 spectra, correlation coefficient of 0.7, and smoothing width of 10 were applied. The generated lists were opened in ProfileAnalysis 2.0 (Bruker Daltonics); group attributes were defined as 1 (Cr(VI)-exposed) and 0 (nonexposed controls). The rectangle bucketing was performed using the following settings: the retention time width 60 s and* m/z* width 1 Da and time range 5.5–44.5 min; sum of buckets option was used for normalization. *t*-test was carried out comparing exposed and nonexposed plants, and minimum fold change was set at 5 and *p* value at 0.01; from this analysis and by additional inspection of intensities (higher than 5 · 10^4^ cps), the precursors list was obtained.

These same raw data were submitted to XCMS, defining a pairwise job. In the instrument selection, UPLC/Bruker QTOF POS was marked, which automatically activated centWave algorithm for FMF. Parameters used for FMF were mass tolerance of 10 ppm between successive measurements, peak width of 5–20 s, S/N threshold of 6, and obiwarp algorithm for retention time alignment (*m/z* width 0.9 s, minimum fraction of samples of 0.5 for group validation). To visualize differences between exposed and nonexposed roots, the cloud plot was obtained applying analogous statistical criteria as those used for ProfileAnalysis. Welch* t*-test was then performed (*p* < 0.01) yielding the list of precursors.

The above two lists of precursors were manually revised leaving only those ions that were obtained by both approaches under criterion of absolute intensity threshold 5 · 10^4^ cps.

Once LC-MS/MS data were acquired in a separate analytical run, molecular formulas were generated with the aid of SIRIUS 3.2 plus CSI:FingerID and MS-Finder tools. Few possible molecular structures with relatively high statistical scores were proposed per each molecular formula and searched in biological databases (ChEBI, HMDB, KEGG, KNApSAcK, MeSH, UNPD, and PubChem), accordingly to the first layers of InChIKey.

## 3. Results and Discussion

The aim of this work was to obtain biologically relevant information on metabolites involved in sunflower response to abiotic stress imposed by Cr(VI). For enhanced reliability, raw capHPLC-ESI-QTOF-MS and MS/MS data were processed using different computational tools, as depicted in Figure 1. Plant selection was based on the demonstrated tolerance of* Helianthus annuus* upon heavy metal stress and its potential feasibility for phytoremediation purposes [11, 12, 25].

### 3.1. Plant Growth and Cr Concentrations in Roots and Shoots

In the preliminary experiments, the following concentrations of chromium in form of Cr(VI) were added to the nutrient solution: 1.0; 5.0; 10; 15; 25; 30; 35; 40; 50 mgCr/L. Growth inhibition was observed in a concentration dependent manner and, starting from the concentration 35 mgCr/L, plants did not grow. For metabolomic study, a dose of 25 mgCr/L was applied; after 10 days' exposure, roots were about 60% shorter as compared to the controls yet chlorophyll levels in leaves were practically not affected. Mean SPAD value for control seedlings was 35.10 ± 0.42 and for the exposed plants it was 32.01 ± 0.57 (chlorophyll meter SPAD-502, Minolta Co. Ltd.). Of note, inhibition of root growth under Cr(VI) stress in plant seedlings has often been reported [26, 27].

Total chromium found in roots was 4.86 ± 0.34 mg/g under exposure to Cr(VI) and 1.33 ± 0.08 *μ*g/g for controls (mean values with respective standard deviations obtained for 4 replicates). In aerial parts, Cr concentrations were substantially lower: 74.5 ± 1.2 *μ*g/g and 1.09 ± 0.03 *μ*g/g for the exposed and control plants, respectively. Similar distribution between roots and shoots has been reported elsewhere [25].

The obtained results confirm suitability of our model for metabolomic study of sunflower response under Cr(VI) stress. It was decided to analyze root extracts, because this morphological part retained chromium and its growth was more markedly inhibited as compared to the aerial part.

### 3.2. LC-MS Analysis and Generation of Precursors List

**Figure 2:**
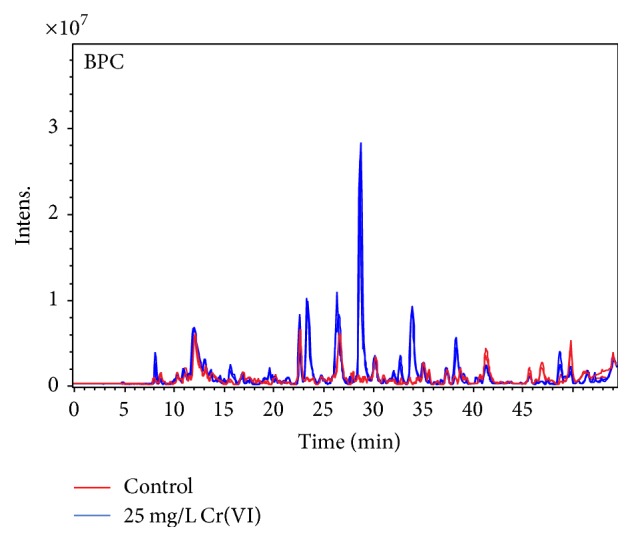
Base peak chromatograms obtained for the root extracts of Cr(VI) exposed plants (blue) and for control, nonexposed plants (red). Two technical replicates are shown for each of the two samples.

**Figure 3:**
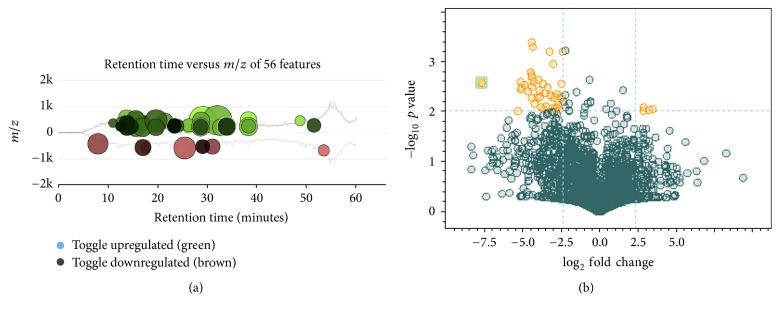
Analyses performed on LC-MS data for generation of precursors list: (a) cloud plot obtained by XCMS with the following settings: intensity > 5 · 10^4^; fold change ≥ 5; *p* ≤ 0.01. (b) Volcano graph obtained by ProfileAnalysis with the following settings: fold change ≥ 5; *p* ≤ 0.01.

In Figure 2, base peak chromatograms obtained for exposed and nonexposed root extracts are presented with substantial differences in the elution profiles clearly observed. In Figure 3, a cloud plot and a volcano graph are presented that were obtained using XCMS platform and ProfileAnalysis software, respectively. During generation of cloud plots, interactive parameters include fold change, *p* value, and intensity threshold whereas ion intensities are not considered in volcano graphs; that is why larger number of molecular features complying with the applied criteria were detected by ProfileAnalysis (Figure 3). On the cloud plot (Figure 3(a)), molecular features that presented higher intensity in the exposed group are marked with green color whereas, in volcano graph (Figure 3(b)), upregulated molecular features are indicated as yellow circles with negative log_2_ fold change values. These features were further inspected to eliminate signals of low intensity (<5 · 10^4^ cps) and to find those that were preselected by two independent tools. As a result, seven molecular features of relatively high intensity, presenting fold change in the range 6–75 between exposed and nonexposed groups at *p* < 0.01, were found. Of note, among 23360 molecular features initially detected, 1930 were preprocessed (*p* ≤ 0.05) and, by application of statistical *t*-test incorporated in two different tools of data analysis, this number was finally reduced to 7. The list of precursors is presented in Table 1 providing the assigned number (ID), retention time,* m/z* value, fold change, and *p* value for each of them.

### 3.3. Annotation of Metabolites with the Aid of SIRIUS and MS-Finder

**Figure 4:**
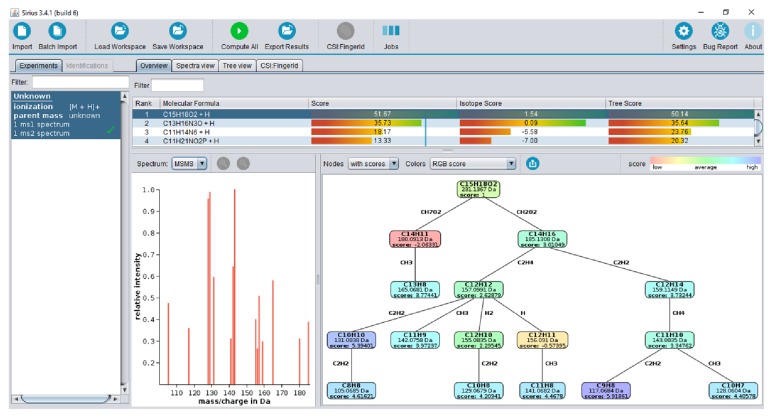
SIRIUS screenshot showing results obtained for the precursor ion* m/z* 231.1367; experimental MS/MS spectrum, fragmentation tree, and the first molecular formulas with respective score values are included.

Spectral data obtained for seven selected compounds (MS and MS/MS) were processed using SIRIUS engine. For each of them, the algorithm computed and ranked all possible molecular formulas. As an example, Figure 4 shows a screenshot with the results obtained for the precursor ion* m/z* 231.1367. It can be observed that the fragmentation tree for C15H28O2 formula was composed of fifteen fragments, eleven of them with the scores in the range 3.01–5.92 (medium to high), two with scores 2.29 and 2.63 (medium), respectively, and only two with negative scores. This formula was found as the first candidate with overall score 51.67. For each predicted formula, possible molecular structures were searched with CSI:FingerID which additionally considers the retention time of given precursor; the proposed structures are annotated with InChIKey code enabling their database search. The three first structures predicted for* m/z* 231.1367 correspond to the secondary metabolites sesquiterpene lactones.

**Figure 5:**
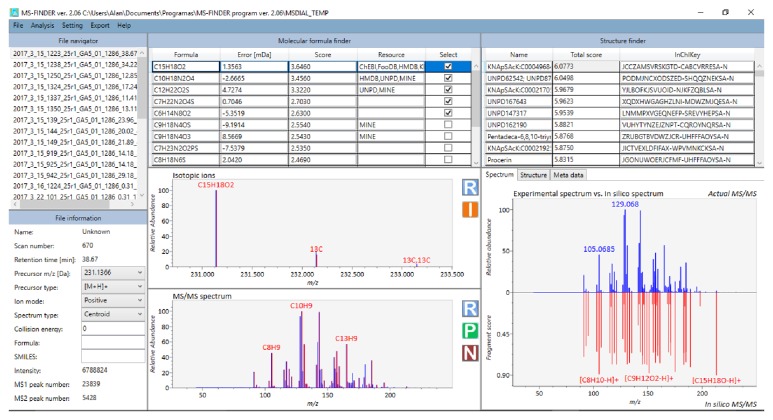
MS-Finder screenshot showing results for the precursor ion* m/z* 231.1367. Experimental (red) and theoretical (blue) isotopic patterns and MS/MS are included together with the fragment score plot. The list of candidate formulas with mass error and score is also presented and, for the selected formula, possible structures found in database search are reported.

In the second approach, MS-Finder was applied to predict molecular formulas and possible structures of seven precursors. Taking this same ion* m/z* 231.1367 and the formula C15H28O2 as an example, the MS-Finder results are presented in Figure 5. Experimental isotopic pattern and MS/MS spectrum soundly matched those calculated in silico; indeed, about 70% of score values assigned for the fragments in experimental MS/MS spectrum were ≥0.5. Using selected databases, resulting InChIKey codes of candidate structures are shown in the screenshot; for* m/z* 231.1367, sesquiterpene structures were suggested (Figure 5).

**Figure 6:**
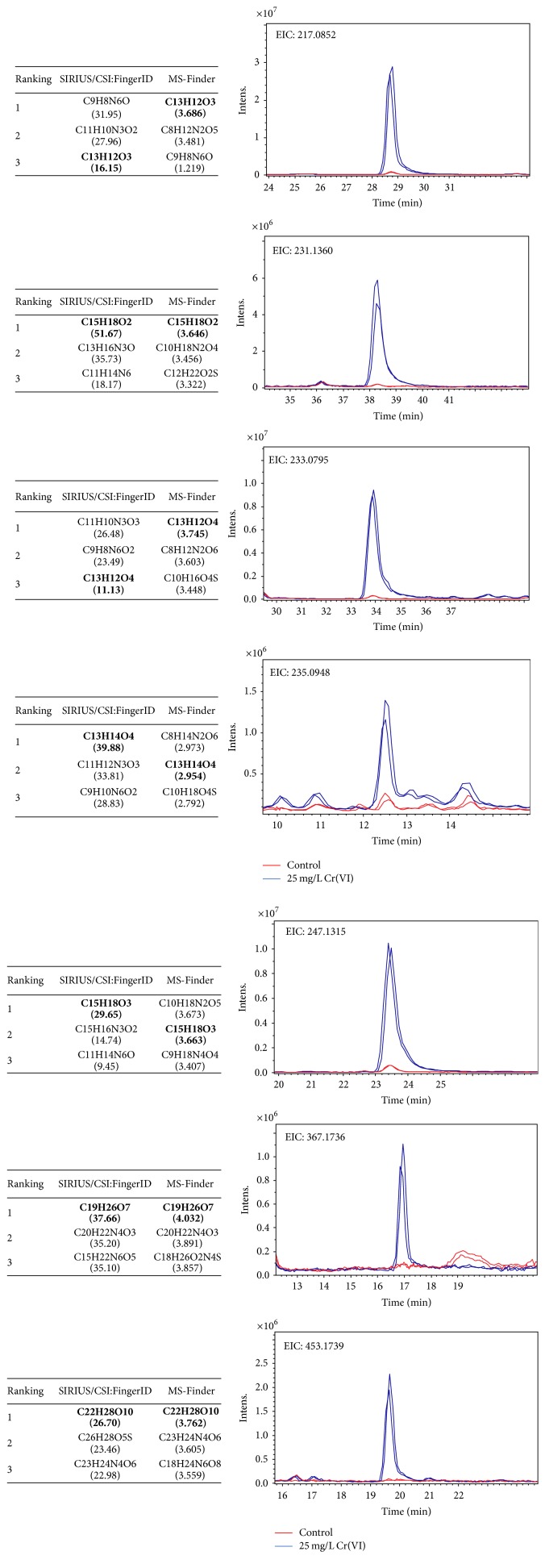
Three first molecular formulas predicted in SIRIUS and MS-Finder for seven precursor ions (score values reported in parentheses, bold font indicates the pondered formula) and the extracted ion chromatograms of these ions acquired for two replicates of exposed and nonexposed roots, respectively (see Table 1 for the description of MF).

LC-MS and MS/MS data obtained for other six precursors were analyzed in the same manner as described above. Three first molecular formulas predicted by two engines were considered and the one which appeared in both lists was pondered as the most reliable. In Figure 6, these formulas are reported together with their score values and the approved one is marked in each case. Additionally, taking the elution region of each precursor, extracted ion chromatograms are presented in Figure 6 for two replicates of the Cr(VI) exposed group and for controls; strong eliciting effect of Cr(VI) is clearly observed (specific fold change values provided in Table 1).

Database search of candidate molecular structures for the pondered formulas was performed from SIRIUS, using CSI:FingerID and directly from MS-Finder. Three first structures found with the aid of two tools are presented in Table 2 together with their InChIKey codes.

### 3.4. Secondary Metabolites Involved in Plant Response under Cr(VI) Stress

As highlighted in the introduction, very few studies have been devoted to the effect of Cr(VI) on plant metabolome [7, 8] and we have found no data regarding* Helianthus annuus*. Experimental evidence obtained in this work did not enable ultimate identification of chemical species affected by Cr(VI); however, candidate structures presented in Table 2 point to the activation of secondary metabolism in sunflower under exposure conditions applied. This finding is supported by earlier studies of the impact of metals/metalloids in different plants [28]. Being a strong oxidant, Cr(VI) causes increased oxidative stress and ROS production [29, 30], thus stimulating cellular signaling pathways of plant defense which potentially includes increased biosynthesis of secondary metabolites [1, 31].

Database search for molecular formula C13H12O3 yielded structures derived from isocoumarin (structures 1–6) that are synthesized in phenylpropanoid pathway. Enhanced production of these compounds had been observed in sunflower under abiotic stress elicited by Cu(II) and sucrose [32]. Isocoumarins are classified as phytoalexins and present a wide range of structure-dependent pharmacological activities. In this regard, the role of hydroxyl groups and alkyl side-chains has been highlighted and, as an example, 3-butylisocoumarins (structures 2 and 6) were studied as antifungal agents [33, 34]. Structures 2 and 6 were found in plants from Asteraceae family (Comprehensive Species-Metabolite Relationship Database KNApSAcK) and structure 4 was in Universal Natural Products Database of Pekin University (UNPD) of natural products whereas 1, 3, and 5 were returned from PubChem search and 3 and 4 from ZINC database.

Six candidates for C15H15O2 formula presented sesquiterpene structures (compounds 7–12, Table 2); four of them (7, 9, 11, and 12) contained *α*-methylene-*γ*-lactone moiety which is known for conferring health relevant biological activity [35, 36]. This group of terpenoids in* Helianthus annuus* has been associated with the defense against pathogens, weeds, and insects [36, 37]. Even though their presence in flowers and aerial parts has been mainly reported [37, 38], participation in rhizosphere interactions was also informed [39]. All six compounds (7–12) were found in UNPD database and, additionally, compounds 11 and 12 were also found in plants from Asteraceae family (KNApSAcK).

Another group of candidate compounds assigned tentatively as sesquiterpenes were those with molecular formula C15H18O3 (precursor ion* m/z* 247.1315). Four structures (25, 27, 29, and 30) were found as sesquiterpene lactones in UNPD and 28 was returned from KNApSAcK search. The first candidate on the list from MS-Finder, phomallenic acid A (UNPD), had a skeleton of acetylenic fatty acids widely occurring in plants and presenting antiherbivory or insecticidal activity [40].

The two first compounds provided by SIRIUS/CSI:MSFinger for formula C13H12O4 shared benzofuran structure (13, 15, UNPD); few hundred benzofurans have been identified in all morphological parts of plants, mainly belonging to Asteraceae family [41]. Compound 14 (UNPD), the first on the MS-Finder list, presented a structure of chromone derivative reported in* Nicotiana tabacum* [42]. Other possible compounds (16, 17) corresponded to isocoumarins and were found in Cardiovascular Disease Herbal Database (CDHD) and UNPD, respectively; chromanone structure was suggested as a candidate 18 (UNPD).

Database search of formula C13H14O4 returned six phenylpropanoids with different structures, all of them included in UNPD. One of these compounds belongs to benzofurans (19) and another to isocoumarins (20); compound 22 was suggested as 1′-acetoxychavicol acetate often reported for its strong antioxidant properties [43].

For formula C19H26O7, two pairs of these same structures were proposed in application of SIRIUS/CSI:FingerID and MS-Finder; certainly, with the increasing* m/z* values (367.1736), MS-based structure prediction becomes more reliable. Candidate compounds 31 and 32 show phenyl glucoside structures (UNPD) whereas 33 and 34 correspond to sesquiterpene lactones (UNPD) particularly abundant and diverse in Asteraceae plants [44]. Increased synthesis of sesquiterpene lactones has been associated with environmental stress, as a part of defensive response against microorganisms and insects, as allopathic agents, and also protecting against abiotic factors [44].

Finally, for the precursor of the highest* m/z* value (453.1739) and molecular formula C22H28O10, also two pairs of candidates were provided independently by SIRIUS/CSI:FingerID and MS-Finder. Structure 36 belongs to the family of iridoid glycosides (davisioside, UNPD, KNApSAcK), chemical compounds found in many plants as secondary metabolites protecting against microbes and insects [45]. Candidate 36 is reported in UNPD as Glochidacuminoside B and suggests phenolic acid-derived glucopyranoside with unknown biological relevance. Candidate 38 is a coumarine derivative (UNPD) identified as tschimganic ester A in* Prangos tschimganica* with demonstrated anti-HIV activity [46].

Overall, considering seven precursor ions and 42 candidate compounds, ten structures corresponded to isocoumarins (26%) and eleven were sesquiterpenes (29%). These groups of secondary metabolites have been reported in Asteraceae family of plants and some of them in* Helianthus annuus* although not within the context of their enhanced synthesis under Cr(VI) stress. In terms of potential biological relevance, data obtained in this study are pioneer and indicate that future study should be focused specifically on the extraction and identification of isocoumarins and sesquiterpenes elicited by Cr(VI). The obtained results might help in better understanding the mechanisms involved in plant defensive response. Most importantly, activation of phenylpropanoid pathway observed in the exposed sunflower roots suggests the enhanced synthesis of lignin to reinforce the cell wall, as often reported in other plants exposed to biotic and abiotic stress [47, 48]. On the other hand, as a prooxidative agent, Cr(VI) promotes generation of reactive oxygen species [9] triggering signaling cascade which involves jasmonate hormone, implicated in regulation of various secondary metabolites, including terpenes [49, 50].

## 4. Conclusions

Abiotic stress imposed by toxic forms of metals/metalloids is a challenging area in metabolomics. In this work, we applied liquid chromatography coupled to high resolution mass spectrometry to gain an insight on the impact that Cr(VI) might have in* Helianthus annuus* roots. Rather than extensive annotation of plant metabolome, the main goal was to ascertain what groups of compounds were mostly affected by the presence of Cr(VI) in hydroponic cultures. For reliable selection of precursor ions, intensity threshold (5 · 10^4^), fold change ≥ 5, and *p* ≤ 0.01 criteria were applied and two computational tools were used (ProfileAnalysis from Bruker and free-access XCMS). For seven selected precursors, molecular formulas were assigned with SIRIUS and MS-Finder algorithms. Three candidates per formula obtained in natural products database search aided by CSI:FingerID and MS-Finder were considered as possible structures. The results obtained point to the increased synthesis of the following secondary metabolites: isocoumarins, sesquiterpenes, and their lactones, benzofurans, glycosides of phenolic compounds. The great majority of candidate compounds had been previously reported in Asteraceae family and some of them in* Helianthus annuus*, but their enhanced synthesis in response to Cr(VI) stress was demonstrated here for the first time. The obtained data allow us to center future study specifically on the identification of isocoumarins and sesquiterpenes elicited by Cr(VI).

## Figures and Tables

**Table 1:** Precursors list and the accepted molecular formulas.

*m/z*	Molecular formula selected	Retention time, min	Fold change	*p*
217.0852	C13H12O3	29.1	33	0.004
231.136	C15H18O2	38.6	34	0.012
233.0795	C13H12O4	34.2	28	0.001
235.0948	C13H14O4	12.9	6	0.001
247.1315	C15H18O3	23.7	19	0.002
367.1736	C19H26O7	17.3	17	0.006
453.1739	C22H28O10	20.0	75	0.004

**Table 2:** Three first molecular structures obtained for the selected formulas of seven precursors using SIRIUS plus CSI:FingerID and MS-Finder, respectively.

*m/z*	Retention time, min	InChIKey	SIRIUS/CSI:FingerID	InChIKey	MS-Finder
217.0852	29.1	GSJJPFOIZRCDPP	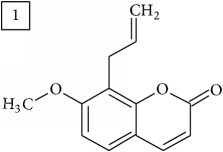	VNEONZFLGNRQJT	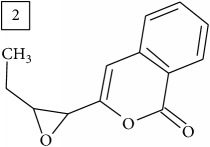
		NYYIJXQZJGQQOP	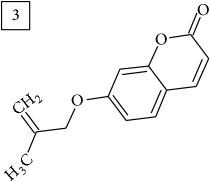	RINHRISDQZMBAU	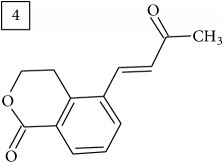
		AGBDCAVWZDTBKU	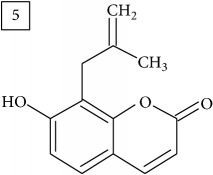	KIVJBPPONQCANS	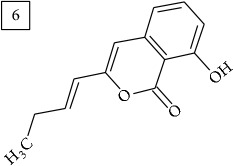

231.1360	38.6	BWRZDLYJNURUHS	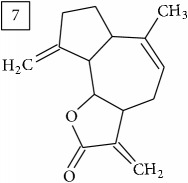	JCCZAMSVRSKGTD	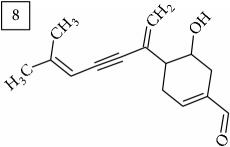
		QLXSYIMXIMTCOX	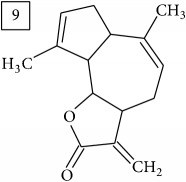	PODMJNCXODSZED	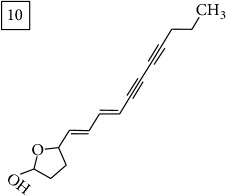
		AVLOGKPJWQCTLP	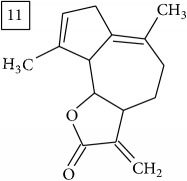	YJLBOFKJSVUOID	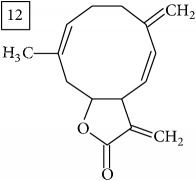

233.0795	34.2	QJIYGTAEIRBFIS	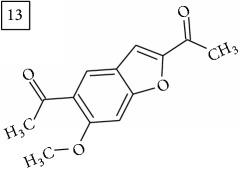	IKOSPDXOTFMSTE	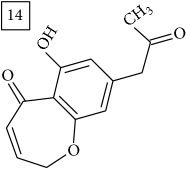
		OHWFUKAVYSSEQD	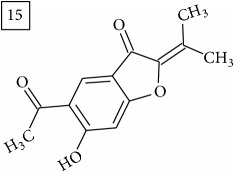	UBVZPPSBGZICHG	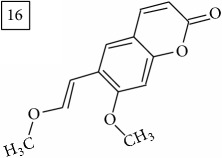
		ZLEKXXXCHQHHFH	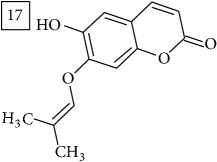	JHELBXAAAYUKCT	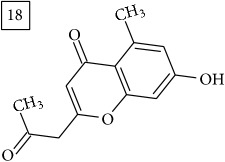

235.0948	12.9	UNXITPCUASJXAZ	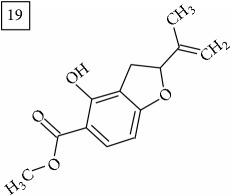	CRXSSRPVDGICML	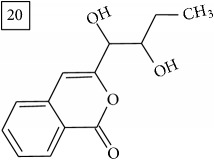
		CIGSWLXZMSXAAE	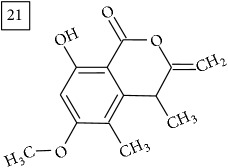	JAMQIUWGGBSIKZ	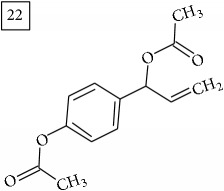
		TXCCGIYIORQRRJ	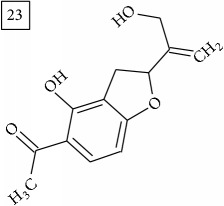	BIUULCNWWFDCPG	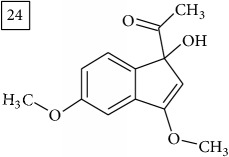

247.1315	23.7	IFXGCKRDLITNAU	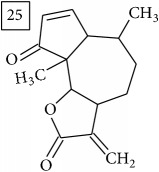	YZCDHRPWTDTXRF	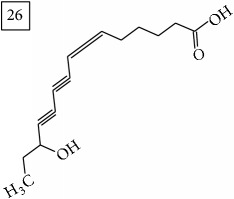
		CHUWSGRBMLBSSX	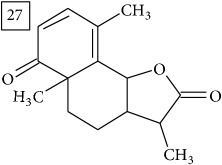	RVSUBOUBGSVZRG	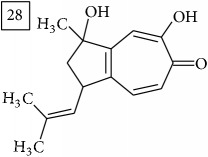
		OSSDUQKWVVZIGP	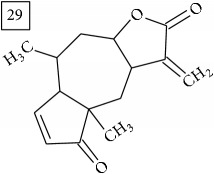	ZLWTZXNAINCRCG	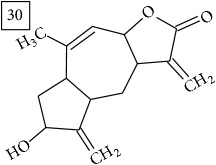

367.1736	38.6	GVWZZKUSNVNWGC	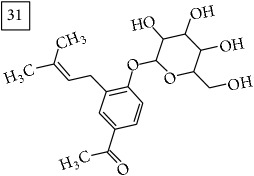	GVWZZKUSNVNWGC	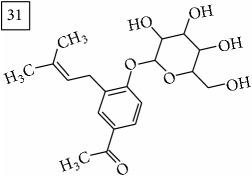
		SPOWTCXDLMRZEG	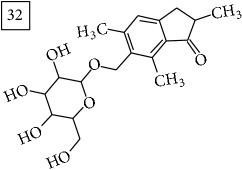	VBHMOJMVNMGQQV	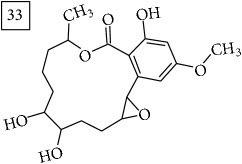
		IFTUAAVSCWBNKT	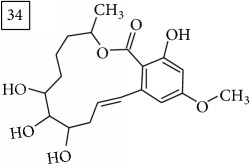	SPOWTCXDLMRZEG	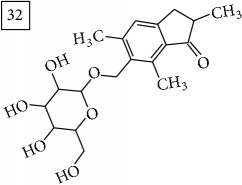

453.1739	20.0	DEDRXCYIYLIKTC	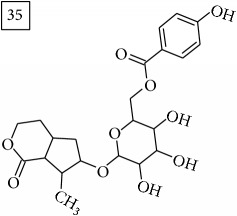	JWZUXOUZLUWWEO	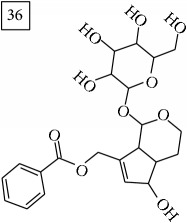
		DERMLURFUNHBPV	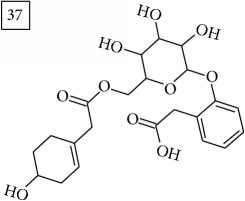	DERMLURFUNHBPV	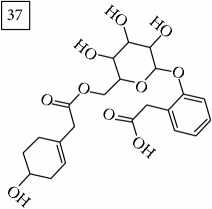
		JWZUXOUZLUWWEO	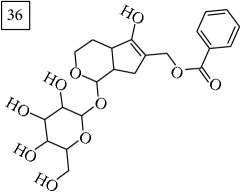	CRZZEGKPXWDZPM